# Integrated Analysis of the Role of Enolase 2 in Clear Cell Renal Cell Carcinoma

**DOI:** 10.1155/2022/6539203

**Published:** 2022-11-14

**Authors:** Jiaren Pan, Yanyan Jin, Xiaoming Xu, Wei Wei, Huafeng Pan

**Affiliations:** Department of Urology, Ningbo No. 2 Hospital, China

## Abstract

Enolase 2 (*ENO2*) has increasingly been documented in multiple cancers in recent years. However, the role of *ENO2* in clear cell renal carcinoma (ccRCC) has not been fully explored. In the present study, open-access data were downloaded from The Cancer Genome Atlas (TCGA), Gene Expression Omnibus (GEO), and the Human Protein Atlas (HPA) databases. All statistical analyses were performed in R and GraphPad Prism 8 softwares. Results showed that *ENO2* was overexpressed in ccRCC tissues and cell lines and correlated with worse clinical features and prognosis. *In vitro* experiments indicated that the inhibition of *ENO2* could hamper the malignant behaviors of ccRCC cells. Gene Set Enrichment Analysis showed that epithelial-mesenchymal transition, KRAS signaling, inflammatory response, angiogenesis, hypoxia, and WNT/*β*-catenin pathways were upregulated in the *ENO2* high-expression group; whereas adipogenesis, DNA repair, and androgen response pathways were downregulated. Immune infiltration analysis indicated that patients with high *ENO2* levels might have higher M2 macrophages and lower *γβ* T cells in the tumor microenvironment, which may account to some extent for the worse prognosis of *ENO2*. Moreover, it was found that patients with low and high *ENO2* expression might be more sensitive to PD-1 therapy and CTLA-4 therapy, respectively. In addition, patients with high *ENO2* expression showed lower sensitivity to common chemotherapy drugs for ccRCC, including axitinib, cisplatin, gemcitabine, pazopanib, sunitinib, and temsirolimus. Overall, these results suggest that *ENO2* is a potential prognosis biomarker of ccRCC and could affect the malignant biological behavior of cancer cells, highlighting its value as a potential therapeutic target.

## 1. Introduction

Renal cell carcinoma (RCC) is one of the most common malignancies in urology, with approximately 250,000 new cases per year globally [1]. It is well-established that clear cell renal cell carcinoma (ccRCC) is the most frequent subtype of RCC [2]. Surgical resection remains the mainstay of treatment for localized ccRCC due to satisfactory prognosis rates [3]. However, about 30% of ccRCC patients develop distant metastasis due to the lack of early symptoms, resulting in a dismal 5-year survival rate [4], emphasizing the need to identify novel and effective molecular markers associated with the early diagnosis and treatment of ccRCC patients.

Enolase 2 (*ENO2*) is a homodimer in mature neurons and cells of neuronal origin that encodes one of the three enolase isoenzymes in mammals [5]. Over the years, studies have demonstrated that ENO2 is widely involved in the physiological and pathophysiological processes of diverse cancers [6]. For example, Wang et al. [7] revealed that nuclear hepatoma-derived growth factor could upregulate the expression of solute carrier family 2 (facilitated glucose transporter), member 4 and *ENO2*, responsible for the activation of glycolysis in gastric cancer cells, which might facilitate the growth of cancer cells and metastasis processes. Liu et al. [8] found that *ENO2* could enhance cell growth, glycolysis, and glucocorticoid resistance of acute lymphoblastic leukemia cells, suggesting that it is a potential therapeutic target. Meanwhile, Zheng et al. [9] showed that insulin-like growth factor 1 could induce deacetylation of *ENO2* in a histone deacetylase 3-dependent manner, further promoting pancreatic cancer metastasis. Besides, Tang et al. [10] reported that kruppel-like factor 12, a transcription factor, could hamper the proliferation of bladder cancer through transcriptional inhibition of *ENO2*. Moreover, Sun et al. [11] found that overexpression of *ENO2* might lead to the enhanced malignant behavior of cancer cells and a worse prognosis, associated with increased glycolysis in papillary renal cell carcinoma. Nevertheless, the role of *ENO2* in ccRCC has hitherto not been fully elucidated.

The rapid development of bioinformatic analysis provides convenience for researchers to identify novel disease biomarkers [12]. In this study, we comprehensively explored the role of *ENO2* in ccRCC through bioinformatics analysis and *in vitro* experiments. Our results showed that *ENO2* was upregulated in ccRCC tissues and was correlated with worse clinical features, including shorter survival time. *In vitro* experiments indicated that the inhibition of *ENO2* could hamper the malignant behaviors of ccRCC cells. Moreover, Gene Set Enrichment Analysis (GSEA) was performed to explore the difference in biological pathways between patients with high and low expression of *ENO2*. The CIBERSORT algorithm was also applied to quantify the immune cell infiltration in ccRCC tissues. Moreover, the bioinformatics analysis suggested that *ENO2* was associated with the glycolysis process and chemosensitivity of ccRCC.

## 2. Methods

### 2.1. Acquisition of Open-Access Data

The open-access data, including transcriptional profile and clinical information, were retrieved from the Cancer Genome Atlas (TCGA) and Gene Expression Omnibus (GEO) databases. In this respect, the transcriptional profile downloaded from the TCGA database was in the “TPM” form. The genomics reference file “Homo_sapiens.GRCh38,gtf” was used for probe annotation. Clinical information downloaded from the TCGA database was in “xml” form and collated using the author's own Perl code. Datasets GSE40435 (platform: GPL10558; 101 pairs of ccRCC tumor and adjacent tissues), GSE53757 (platform: GPL570; 72 pairs of ccRCC tumor and adjacent tissues), and GSE105261 (platform: GPL10558; nine normal renal tissues, nine primary ccRCC tissues, and 26 metastatic ccRCC tissues) were obtained from the GEO database. Before analysis, the “limma” and “affy” packages were utilized for data preprocessing of the individual cohort, including probe annotation, missing value completion, normalization and correction. The representative immunohistochemistry (IHC) pictures in ccRCC tumor and normal renal tissues were obtained from The Human Protein Atlas database (HPA; https://www.proteinatlas.org/). Finally, the genomics of drug sensitivity in cancer (GDSC) database was utilized to perform drug sensitivity analysis to explore the underlying effect of *ENO2* on ccRCC chemotherapy (http://www.cancerrxgene.org/).

### 2.2. Analysis of Differentially Expressed Genes (DEGs) and Construction of a Protein-Protein Interaction (PPI) Network

The “Limma” R package was applied to identify DEGs between ccRCC and normal renal tissues, with the threshold of |logFC| > 1 and *P* value <0.05 as the cutoffs. A PPI network was constructed based on the STRING website (https://cn.string-db.org/) and then visualized in Cytoscape_v3.7.2 software. The relative importance of each node was calculated using the CytoHubba plug-in of Cytoscape.

### 2.3. Clinical Correlation and Prognosis Analysis

The clinical features of ccRCC patients in TCGA, including survival status, age, gender, and clinical stage, were collated and combined with *ENO2* expression data. Patients were stratified into low and high expression groups based on the median cutoff, and then the R package “survival” was applied to assess the prognosis of groups with different expression levels of *ENO2*.

### 2.4. Pathway Enrichment Analysis

GSEA analysis was utilized to explore the biological difference between high and low *ENO2* expression patients, which was completed using the “clusterProfiler” and “fgsea” packages in R software. Notably, the hallmark was defined as the reference dataset. The top ten downregulated and upregulated pathways were selected for visualization. Gene ontology (GO) and Kyoto Encyclopedia of Genes and Genomes (KEGG) enrichment analyses were then performed using the R package “clusterProfiler”.

### 2.5. Immune Infiltration and Immunotherapy Response Analysis

The CIBERSORT algorithm was applied to explore the difference in immune infiltration in tissues with high and low *ENO2* expression to quantify the relative proportions of 22 types of infiltrating immune cells. Tumor Immune Dysfunction and Exclusion (TIDE) and the submap algorithm were then used to assess the efficacy of immunotherapy.

### 2.6. Cell Lines and Quantitative Real-Time PCR (qPCR)

Human renal tubular epithelial cell line (HK-2) and human ccRCC cell lines (ACHN, 786-O, Caki-1) were purchased from the cell bank of the Chinese Academy of Sciences. Total RNA was extracted from cells using the TRIzol method, followed by reverse transcription to cDNA for further experiments. qPCR was performed using a PCR kit (Takara, Kyoto, Japan) according to the manufacturer's protocol. SYBR Green system was then used to detect the amplification products. The following primers were used: *ENO2*: forward, 5′-CATCTGTGATGGGAGCGTCA-3′; reverse, 5′-TGGGACAAGAGCAAAGCACA-3′ and GAPDH: forward, 5′-CTGGGCTACACTGAGCACC-3′; reverse, 5′-AAGTGGTCGTTGAGGGCAATG-3′.

### 2.7. Cell Transfection

Lipofectamine 2000 transfection kits (Invitrogen) were used for cell transfection. Notably, the shRNA and control plasmid of *ENO2* were obtained from Shanghai Ji Kai Chemical Technology. The shRNA target sequences used were as follows: siRNA1; 5′-CAAGGGAGTCATCAAGGACAA-3′, siRNA2; 5′-CGCCTGGCTAATAAGGCTTTA-3′, and siRNA3; 5′-GTGTATTTATTTATTTATTTA-3′.

### 2.8. Western Blot

A total protein extraction kit (Beyotime, Jiangsu, China) was used to extract the total protein following the manufacturer's protocol. The SDS–polyacrylamide gel electrophoresis (SDS-PAGE) glue was utilized for the western blot based on the standard process. The primary antibody of ENO2 (1 : 5000) and GAPDH (1 : 10000) were purchased from Proteintech.

### 2.9. CCK8 Assay

A CCK8 kit (Dojindo, Shanghai, China) was used to perform the CCK8 assay following the manufacturer's protocol. Briefly, cells were resuspended and inoculated into a 96-well plate at a density of 1 × 10^4^ cells per well. The CCK8 reagent was added to the plate, followed by incubation at 37°C for 2 h. Finally, the absorbance was measured at OD 450 nm using an ELISA plate reader (BioTek, Winooski, VT, USA).

### 2.10. Colony Formation Assay

Cells were resuspended and inoculated into a six-well plate at a density of 500 cells per well. Cells were then maintained for 14 days in an incubator. Finally, cells were fixed with 4% paraformaldehyde and stained with crystal violet.

### 2.11. Transwell Assay

A transwell chamber was used to divide the 24-plate well into two chambers: the upper chamber and the lower chamber. Next, 3 × 10^4^ ccRCC cells and 250 *μ*L medium with 10% bovine serum albumin (BSA) were added to the upper chamber, whereas 500 *μ*L medium without BSA was added to the lower chamber. After 12 h, cells were fixed with 4% paraformaldehyde and stained with crystal violet.

### 2.12. Xenograft Nude Mouse Model

We purchased five-week-old male BALB/c nude mice for the xenograft model test. Briefly, 5 × 10^5^ 786-O cells in the control and inhibition groups were subcutaneously inoculated into the back of mice. All the mice were sacrificed for 20 days and weighed.

### 2.13. Immunohistochemistry

Paraffin-embedded 5 *μ*m thick sections of mice tumor tissues were cut. Immunohistochemistry was performed using the *ENO2* primary antibody (6F8G3, Proteintech) based on the standard process. Positive expression was observed as cytoplasmic yellow-, brown-, or tan-colored staining.

### 2.14. Statistical Analyses

All statistical analyses were performed using R and GraphPad Prism 8 softwares. A *P* value less than 0.05 was statistically significant. All experiments were replicated at least three times. The Student *t*-test was used to compare the difference of data conforming to a normal distribution, whereas the Kruskal–Wallis test was used for data with a nonparametric distribution.

## 3. Results

### 3.1. Identification of *ENO2* through Bioinformatics Analysis

**Figure 1:**
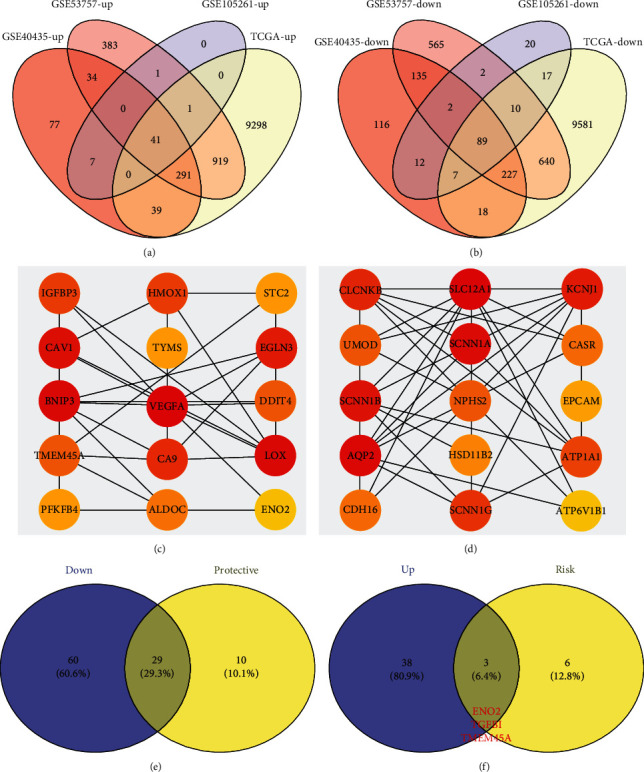
The identification process of *ENO2* as the target gene in ccRCC. Notes: (a) intersection of four individual databases (GSE40435, GSE53757, GSE105261, and TCGA) identified 41 genes showing an upregulated pattern in ccRCC tissues. (b) Intersection of the four individual databases (GSE40435, GSE53757, GSE105261, and TCGA) identified 89 genes showing a downregulated pattern in ccRCC tissues. (c) Top 20 key nodes of the 41 upregulated genes based on PPI network. (d) Top 20 key nodes of the 89 downregulated genes based on PPI network. (e) Among the 89 downregulated genes, 29 genes were protective factors. (f) Among the 41 upregulated genes, three genes were risk factors, including *ENO2, TGFBI*, and *TMEM45A*.

We first identified DEGs based on the transcriptional profile data of ccRCC and normal renal tissues in multiple public databases (GSE40435, GSE53757, GSE105261, and TCGA). Results revealed that 41 upregulated and 89 downregulated DEGs exhibited similar expression patterns in the four datasets (Figures 1(a) and 1(b)). Next, the top 20 important nodes in the 41 upregulated and 89 downregulated DEGs were visualized, which might play key roles in ccRCC tumorigenesis (Figures 1(c) and 1(d)). Univariate Cox regression analysis was then performed to identify prognosis-related DEGs. It was found that 29 DEGs that were downregulated in ccRCC were protective factors (HR < 1; P < 0.05) (Figure 1(e)). Meanwhile, three DEGs that were overexpressed in ccRCC were risk factors (HR > 1; P < 0.05), including *ENO2*, transforming growth factor, beta-induced (*TGFBI*), and transmembrane protein 45A (*TMEM45A*) (Figure 1(f)). Moreover, *ENO2* was the key node in the PPI network of upregulated DEGs and was, thus, selected for further analysis.

### 3.2. *ENO2* Was Upregulated in ccRCC

**Figure 2:**
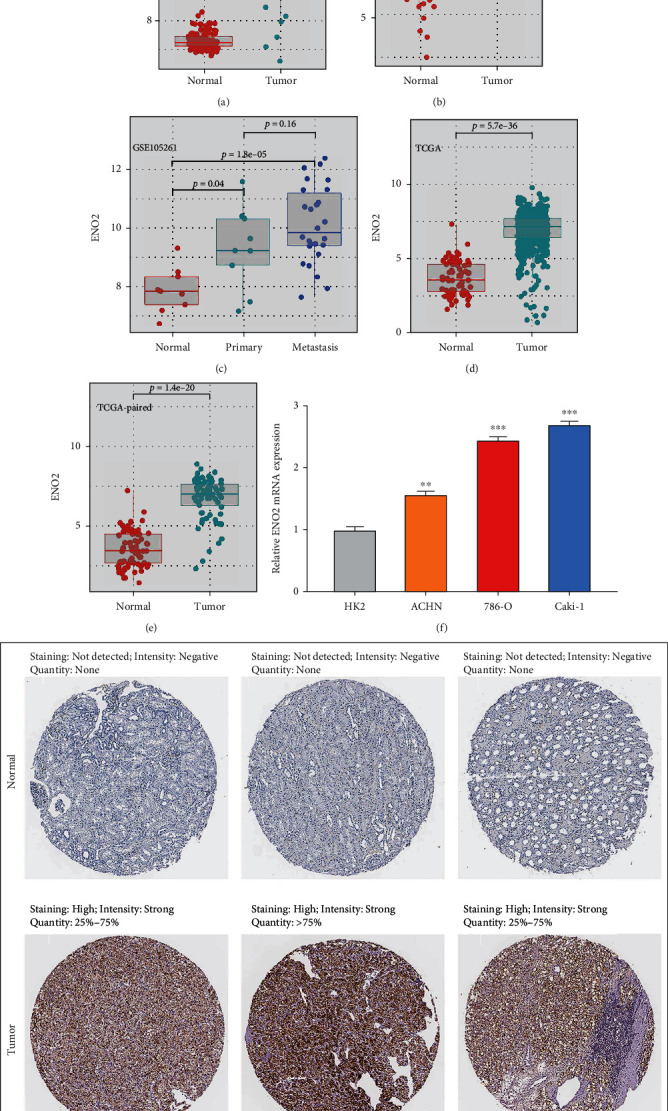
*ENO2* was overexpressed in ccRCC tissues and cell lines. Notes: (a, b) *ENO2* was upregulated in ccRCC tissues based on the GSE40435 and GSE53757 datasets. (c) *ENO2* was also overexpressed in metastatic ccRCC tissues compared to the primary ccRCC tissues. (d, e) *ENO2* was upregulated in ccRCC tissues based on the TCGA database. (f) *ENO2* was upregulated in ccRCC cell lines compared to the normal HK-2 cell line. (g) Representative IHC images obtained from the HPA database showed an overexpressed pattern of *ENO2* in ccRCC tissues.

Datasets GSE40435, GSE53757, and GSE105261 showed that ccRCC tissues had a higher *ENO2* RNA expression level than normal renal tissues (Figures 2(a) and 2(b)). It was also found that metastasis ccRCC tissues exhibited higher *ENO2* expression than the primary ccRCC tissues, indicating that *ENO2* may be associated with distant metastasis (Figure 2(c)). Similar results were observed by analysis in the TCGA database (Figures 2(d) and 2(e)). Furthermore, we explored the expression of *ENO2* in ccRCC cell lines. Results showed that *ENO2* was overexpressed in ccRCC cell lines compared to normal HK-2 cells (Figure 2(f)). The representative IHC images from the HPA database indicated higher *ENO2* protein expression in ccRCC tissues (Figure 2(g)).

### 3.3. *ENO2* Was Associated with worse Clinical Features in ccRCC Patients

**Figure 3:**
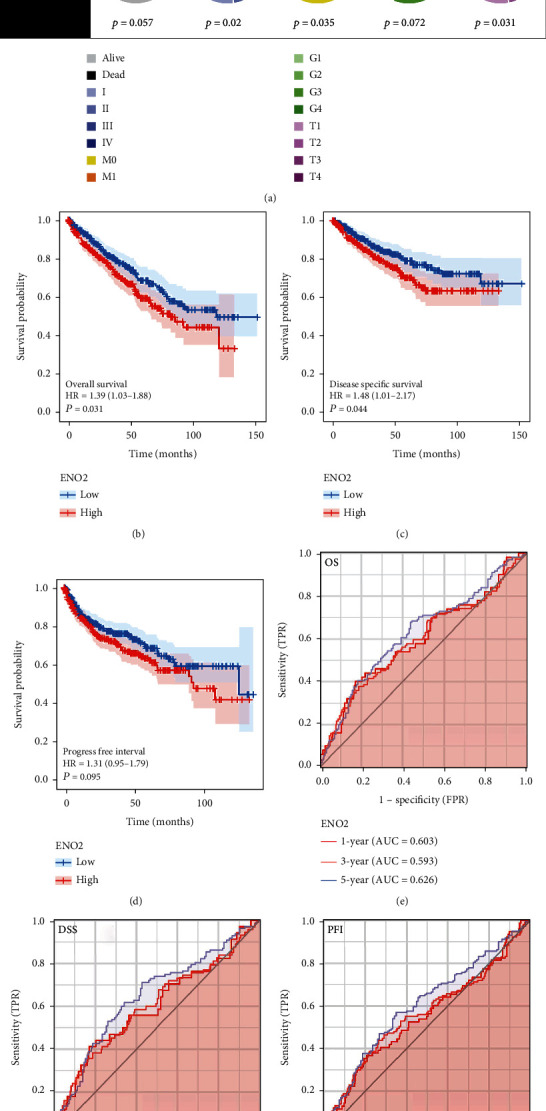
*ENO2* was associated with worse clincical features and prognosis of ccRCC. Notes: (a) The ccRCC patients with higher *ENO2* expression were significantly correlated with worse clinical features, including clinical stage and T and M stages. (b–d) The ccRCC patients with higher *ENO2* expression tended to have a shorter OS, DSS, and PFI. (e–g) The ROC curve of OS, DSS, and PFI.

Next, we explored the association between *ENO2* and the clinical features of ccRCC.  Table 1 shows the detailed clinical features of ccRCC patients in TCGA. We found that *ENO2* was correlated with worse Tumor (T) and Metastasis (M) stages according to the AJCC staging system (Figure 3(a)). The prognosis value of *ENO2* was also explored with the overall survival (OS), disease-specific survival (DSS), and progression-free survival (PFI) set as endpoints. Results showed that high *ENO2* expression correlated with worse OS and DSS (Figures 3(b) and 3(c)). Despite not being statistically significant, different KM curves were obtained for patients with high and low *ENO2* expression, indicating that *ENO2* could affect the PFI of ccRCC patients (Figure 3(d)). Moreover, receiver operating characteristic (ROC) curves showed a good prediction efficiency of *ENO2* on patients prognosis (Figure 3(e): 1-year AUC = 0.603, 3-year AUC = 0.593, and 5-year AUC = 0.626; Figure 3(f): 1-year AUC = 0.601, 3-year AUC = 0.601, and 5-year AUC = 0.647; and Figure 3(g): and 1-year AUC = 0.554, 3-year AUC = 0.563, and 5-year AUC = 0.605).

### 3.4. Inhibition of ENO2 Could Hamper Malignant Behaviors of ccRCC Cell

**Figure 4:**
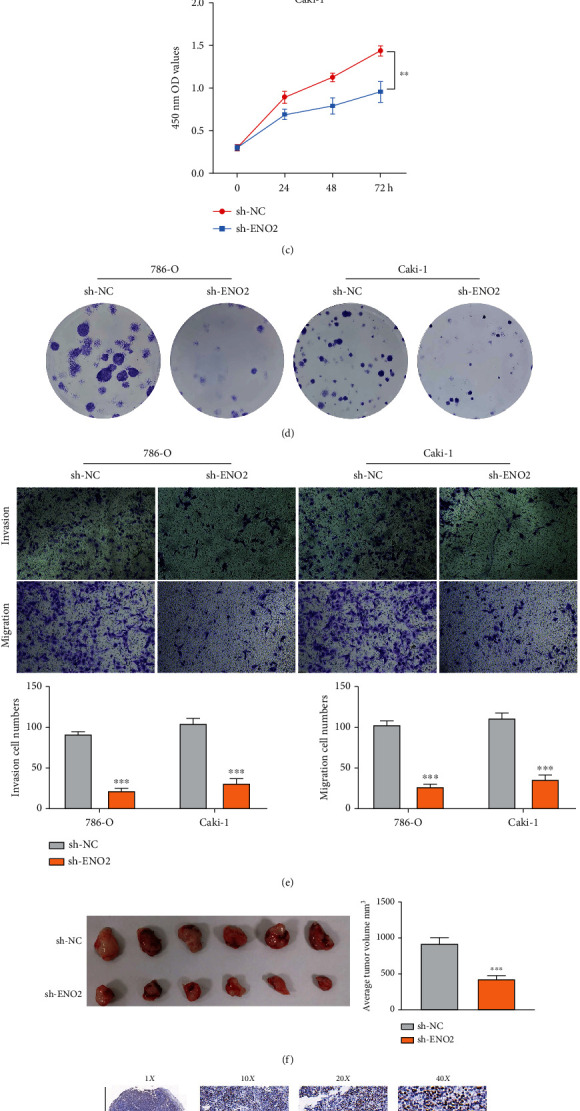
Inhibition of *ENO2* can hamper malignant behaviors of ccRCC cell. Notes: (a) The knockdown efficiency of three designed shRNA were evaluated using qPCR assay, with results showing that shENO2#2 was the best. (b–d) CCK8 and colony formation assays indicated that *ENO2* could significantly promote proliferation of ccRCC cells. (e) Transwell assay showed that *ENO2* significantly promoted invasion and migration of ccRCC cells. (f) *In vivo* experiments showed that the knockdown of ENO2 remarkably lowered the growth of tumor in mice. (g) Immunohistochemistry showed a higher ENO2-positive staining level in the mice tumor tissue inoculated with sh-NC cells.

To explore the biological effect of *ENO2* in ccRCC, we further knocked down *ENO2* in 786-O and Caki-1 cells (Figure 4(a)). The knockdown efficiency of *ENO2* in the protein level was also validated using the western blot assay (Figure S1). The sh-RNA#2 was used for further experiments for its highest knockdown efficiency. CCK8 and colony formation assay was then performed to evaluate the cell proliferation ability. The CCK8 assay showed that the cells in the ENO2 knockdown group had a lower OD value at 450 nm compared to the cells in the control group, indicating that inhibition of *ENO2* could significantly reduce the proliferation ability of ccRCC cells (Figures 4(b) and 4(c)), consistent with findings in the colony formation assay (Figure 4(d)). The transwell assay showed that the knockdown of *ENO2* could significantly hamper the invasion and migration ability of ccRCC cells (Figure 4(e)). *In vivo* experiments showed that the inhibition of *ENO2* significantly hampered the growth of tumors in mice (Figure 4(f)). Also, immunohistochemistry indicated a higher *ENO2*-positive staining level in the mice tumor tissue inoculated with sh-NC cells (Figure 4(g)).

### 3.5. Pathway Enrichment and Immune Infiltration Analysis of *ENO2*

**Figure 5:**
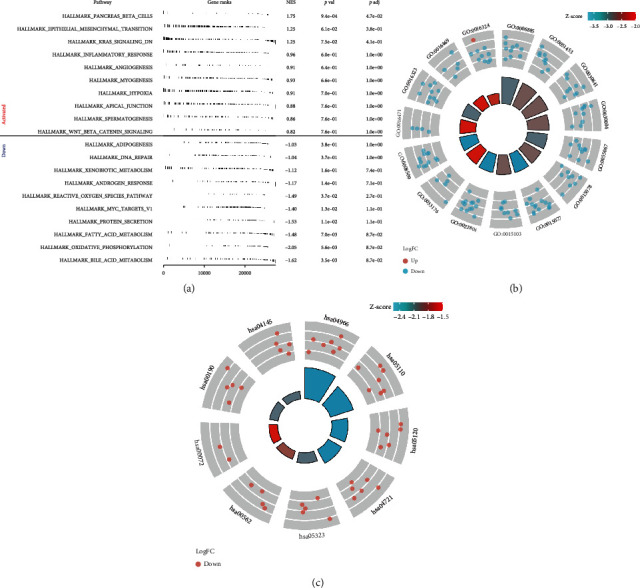
Pathway enrichment of *ENO2* in ccRCC. Notes: (a) GSEA analysis of *ENO2* in ccRCC. (b, c) GO and KEGG analyses of ENO2 in ccRCC.

**Figure 6:**
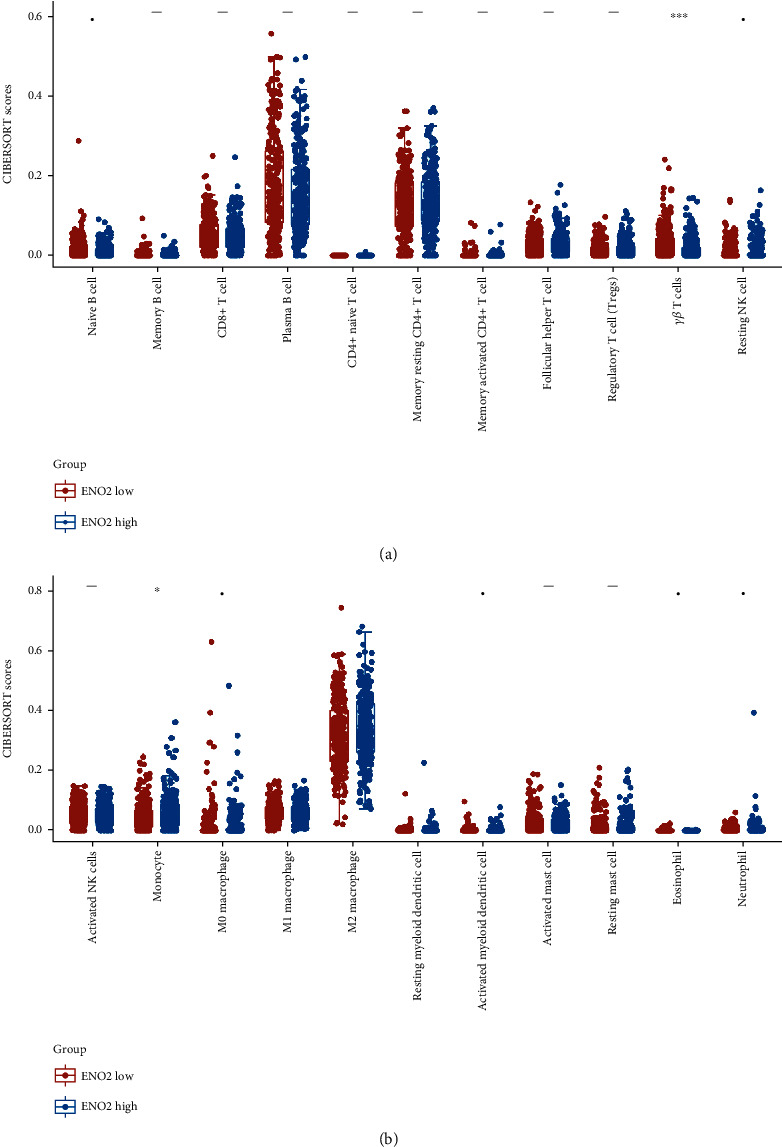
Immune infiltration analysis of *ENO2* in ccRCC. Notes: (a, b) CIBERSORT algorithm was used to quantify the immune infiltration difference in low and high *ENO2* expression patients.

Furthermore, we performed GSEA and CIBERSORT analyses to explore the underlying mechanism of *ENO2* in ccRCC. GSEA results indicated that epithelial-mesenchymal transition (EMT), KRAS signaling DN, inflammatory response, angiogenesis, hypoxia, and WNT/*β*-catenin pathways were upregulated in patients with high expression of *ENO2* (Figure 5(a)). Meanwhile, the pathways of adipogenesis, DNA repair, and androgen response were downregulated in the high-expression group (Figure 5(b)). GO and KEGG analyses showed that *ENO2* was significantly enriched in regulation of pH, inorganic cation homeostasis, and cell transmembrane transport (Figures 5(b) and 5(c) and Table 2). Moreover, immune infiltration analysis showed higher M2 macrophages and lower *γβ* T cells in the tumor microenvironment in patients with high *ENO2* expression (Figures 6(a) and 6(b)).

### 3.6. *ENO2* Is Associated with Immunotherapy and Chemosensitivity in ccRCC

**Figure 7:**
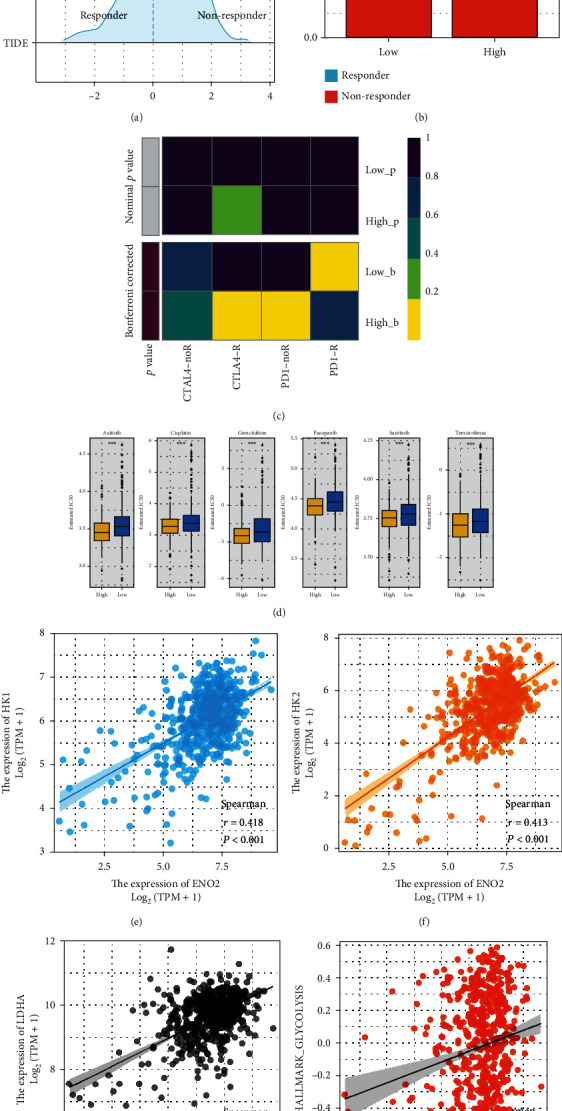
*ENO2* was associated with immunotherapy and chemosensitivity in ccRCC. Notes:(a) TIDE score < 0 were defined as immunotherapy responders, whereas TIDE score > 0 were defined as immunotherapy nonresponders. (b) A higher percentage of immunotherapy responders in the low *ENO2* expression group compared to the high ENO2 expression group. (c) Submap algorithm was performed to explore the difference in the immunotherapy response rate between low and high *ENO2* expression patients, (d) Analysis of the GDSC database showed that patients with high ENO2 expression exhibited lower chemosensitivity to common chemo drugs for ccRCC. (e–g) The correlation between *ENO2* and glycolysis-related genes, *HK-1, HK-2*, and *LDHA*. (h) The correlation between *ENO2* and glycolysis activity.

Given that cancer immunotherapy is reportedly a promising therapeutic choice for ccRCC, we explored whether *ENO2* could affect the immunotherapy sensitivity of ccRCC patients. Therefore, TIDE analysis was performed based on the expression profile data of ccRCC patients, in which patients with TIDE score < 0 were defined as immunotherapy responders, and patients with TIDE score > 0 were defined as immunotherapy nonresponders (Figure 7(a)). We observed a higher percentage of immunotherapy responders in the low *ENO2* expression group compared to the high *ENO2* expression group (Figure 7(b), 42.6% vs. 27.9%, *P* < 0.001). Subgroup analysis showed that patients with low *ENO2* expression might be more sensitive to PD-1 therapy, while patients with high *ENO2* expression might be more sensitive to CTLA-4 therapy (Figure 7(c)). Next, we explored the potential association between *ENO2* and target drugs of ccRCC. Interestingly, analysis of the GDSC database showed that patients with high *ENO2* expression exhibited lower chemosensitivity to common chemo drugs for ccRCC, including axitinib, cisplatin, gemcitabine, pazopanib, sunitinib, and temsirolimus (Figure 7(d)). It has been shown that ENO2 could induce cell glycolysis in multiple cancers [8, 11]. Given that glycolysis promotes cancer in most solid tumors, we speculate that *ENO2* might exert a cancer-promoting effect in ccRCC by regulating glycolysis [13]. We further explored the correlation between *ENO2* and several key molecules participating in glycolysis, hexokinase 1 (*HK1*), hexokinase 2 (*HK2*), and lactate dehydrogenase A (*LDHA*). Results showed a positive correlation between *ENO2* and these molecules (Figure 7(e); *HK1*: *r* = 0.418, *P* < 0.001; Figure 7(f); *HK2*: *r* = 0.413, *P* < 0.001; and Figure 7(g); *LDHA*: *r* = 0.339, *P* < 0.001). Moreover, a positive correlation was found between *ENO2* and quantified glycolysis activity (Figure 7(h); *r* = 0.153, P < 0.001).

## 4. Discussion

Notwithstanding that significant scientific inroads have been achieved in recent years, RCC remains a threatening public health issue globally, and ccRCC is the most common subtype [14]. Although surgery can effectively improve the prognosis of localized ccRCC patients, the five-year survival rate for patients with advanced stages remains poor. Therefore, there is an urgent need to identify new biomarkers associated with the diagnosis and treatment of ccRCC patients.

To the best of our knowledge, this is the first study to comprehensively explore the role of *ENO2* in ccRCC. We found that *ENO2* was highly expressed in ccRCC and correlated with worse clinical features in patients. Moreover, *in vitro* experiments found that *ENO2* the inhibition of ENO2 could hamper the malignant behaviors of ccRCC cell. GSEA and immune infiltration analysis were also conducted to explore the biological difference between patients with high and low *ENO2* expression. Moreover, *ENO2* was associated with upregulated glycolysis activity and chemosensitivity of ccRCC. Our study refined the effect network of *ENO2* in cancer and concluded that *ENO2* is an underlying therapeutic target for ccRCC.

Pathway enrichment analysis is a powerful tool used to identify the underlying mechanisms of specific genes. In the present study, GSEA showed that the EMT process, KRAS signaling, inflammatory response, angiogenesis, and Wnt/*β*-catenin signaling pathways were significantly enriched in the high *ENO2* expression group. It has been reported that EMT is a process during which epithelial cells acquire mesenchymal features, making cancer cells more invasive [15]. Fang et al. [16] found that huaier polysaccharide could hamper ccRCC progression by inhibiting the EMT process, thereby enhancing sunitinib therapeutic effects. In addition, Gorka et al. [17] demonstrated that zinc finger CCCH-type containing 12A could suppress the Wnt/*β*-catenin signaling pathway and modulate the EMT process to influence ccRCC progression. Moreover, KRAS signaling plays a cancer-promoting role in multiple cancers [18]. For instance, KRAS signaling is a critical driver in pancreatic cancer [19]. Wang et al. [20] revealed that miR-216b could downregulate KRAS expression at the post-transcriptional level in ccRCC and inhibit the proliferation and invasion of ccRCC cells. It has also been reported that angiogenesis is involved in the distant metastasis of cancers [21]. Cao et al. [22] showed that decylubiquinone could hamper the proliferation and metastasis of breast cancer cells by inhibiting angiogenesis mediated by the reactive oxygen species/p53/brain angiogenesis inhibitor 1 signaling pathway. G. Wang et al. [23] found that lncRNA MAGI2-AS3 could inhibit angiogenesis through interaction with transcription factor aminoacylase 1, further hampering ccRCC progression. Moreover, Q. Wang et al. [24] revealed that the NLR family, CARD domain containing 5 (NLRC5), could regulate ccRCC proliferation, migration, and invasion by modulating the Wnt/*β*-catenin signaling pathway. These pathways are potential biological pathways associated with *ENO2*. The oncogenetic effect of *ENO2* might be achieved by affecting the activity of the above pathways. Future studies on *ENO2* should focus on interfering with the above pathways to identify the downstream mechanism.

The tumor immune microenvironment has an important impact on the malignant behavior of cancer cells through cell interaction [25]. Herein, immune infiltration analysis showed that *ENO2* was positively correlated with M2 macrophages and negatively correlated with *γβ* T cells. Over the years, M2 macrophages have been reported to exert an immunosuppressive function in multiple cancers [26]. For example, Xie et al. [27] demonstrated that M2 macrophages could secrete chemokine (C-X-C motif) ligand 13 to promote ccRCC migration, invasion, and EMT. Martínez et al. [28] showed that overexpression of bone morphogenetic protein 4 could induce polarization of M2 macrophages, thereby facilitating proliferation, invasion, and migration of bladder cancer cells. Besides, Xu et al. [29] found that hexokinase 3 dysfunction could promote tumorigenesis and immune escape by upregulating the infiltration of monocytes/macrophages into the ccRCC microenvironment. Until now, the relationship between *ENO2* and immune infiltration has been largely understudied. Overall, the correlation between *ENO2* and specific immune cells indicates its underlying effect on the tumor microenvironment.

Furthermore, our results showed that ccRCC patients with higher *ENO2* expression levels might be associated with upregulated glycolysis activity. Meanwhile, patients with high *ENO2* expression might have lower chemosensitivity to the common chemotherapy drugs of ccRCC, including axitinib, cisplatin, gemcitabine, pazopanib, sunitinib, and temsirolimus. Glycolysis has been widely reported to facilitate ccRCC progression. Fang et al. [30] found that succinate dehydrogenase complex, subunit B, and iron-sulfur (Ip) could inhibit ccRCC progression by suppressing glycolysis. In addition, Chen et al. [31] demonstrated that the non-POU domain containing octamer-binding-transcription factor binding to IGHM enhancer 3 fusion promotes aerobic glycolysis and angiogenesis by targeting hypoxia-inducible factor 1, alpha subunit in RCC. The positive correlation with glycolysis can be attributed to some extent to the cancer-promoting effect of *ENO2* in ccRCC. Nowadays, much emphasis has been placed on individualized medical treatment. Interestingly, detecting *ENO2* expression could indicate the difference in sensitivity of patients for specific therapy options. Moreover, for advanced patients with high *ENO2* expression, CTLA-4 immunotherapy might be more appropriate than PD-1 therapy. Meanwhile, patients with high *ENO2* expression tend to have a worse prognosis and higher potential for distant metastasis. For this patient population, a comprehensive postoperative follow-up is warranted.

Indeed, some limitations were present in this study. Firstly, bioinformatics data analyzed in the present study was obtained from western patients. Therefore, race bias was inevitable, which might affect the reliability of our findings to a certain extent and their generalization to non-Western populations. Moreover, the clinical data obtained from TCGA was incomplete. For example, data on the M stage of many patients was unavailable, which might affect the robustness of our conclusion to some degree.

## 5. Conclusion

Overall, this study revealed that *ENO2* was overexpressed in ccRCC tissues and cell lines. In addition, *ENO2* could significantly affect ccRCC progression and was associated with worse clinical features. GSEA analysis indicated that *ENO2* might be involved in activating several oncogenic pathways. Immune infiltration analysis showed that patients with high *ENO2* expression might have higher M2 macrophages and lower *γβ* T cells and monocytes in the tumor microenvironment. Moreover, bioinformatics analysis indicated that *ENO2* was associated with the glycolysis process and chemosensitivity of ccRCC, thus, making it a potential therapeutic target for this patient population.

## Figures and Tables

**Table 1:** Clinical features of patients included in our analysis.

Characteristic	Number	Percentage (%)
Age		
≤60	266	49.5
>60	271	50.5
Gender		
Female	191	35.6
Male	346	64.4
Grade		
G1	14	2.6
G2	230	42.8
G3	207	38.5
G4	78	14.5
Unknown	8	1.5
Stage		
Stage I	269	50.1
Stage II	57	10.6
Stage III	125	23.3
Stage IV	83	15.5
Unknown	3	0.1
T-classification		
T1	275	51.2
T2	69	12.8
T3	182	33.9
T4	11	2.0
N-classification		
N0	240	44.7
N1	17	3.2
Unknown	280	52.1
M-classification		
M0	426	79.3
M1	79	14.7
Unknown	32	6.0

**Table 2:** Top terms of GO and KEGG analyses of ENO2.

Pathway enrichment	Pathway ID	Description
GO	GO:0016324	Apical plasma membrane
	GO:0006885	Regulation of pH
	GO:0051453	Regulation of intracellular pH
	GO:0030641	Regulation of cellular pH
	GO:0030004	Cellular monovalent inorganic cation homeostasis
	GO:0055067	Monovalent inorganic cation homeostasis
	GO:0015078	Proton transmembrane transporter activity
	GO:0015077	Monovalent inorganic cation transmembrane-transporter activity
	GO:0015103	Inorganic anion transmembrane-transporter activity
	GO:0022804	Active transmembrane-transporter activity
	GO:0033176	Proton-transporting V-type ATPase complex
	GO:0008509	Anion transmembrane-transporter activity
	GO:0016471	Vacuolar proton-transporting V-type ATPase complex
	GO:0016323	Basolateral plasma membrane
	GO:0016469	Proton-transporting two-sector ATPase complex
KEGG	Hsa04145	Phagosome
	Hsa04966	Collecting duct acid secretion
	Hsa05110	Vibrio cholerae infection
	Hsa05120	Epithelial cell signaling in helicobacter pylori infection
	Hsa04721	Synaptic vesicle cycle
	Hsa05323	Rheumatoid arthritis
	Hsa00562	Inositol phosphate metabolism
	Hsa00072	Synthesis and degradation of ketone bodies
	Hsa00190	Oxidative phosphorylation

## Data Availability

Detailed information is available from the corresponding author upon reasonable request.

## Supplementary Materials

Supplementary Materials Figure S1. Western blot was used to validate the knockdown efficiency of *ENO2.*

## References

[1] Jonasch E., Walker C. L., Rathmell W. K. (2021). Clear cell renal cell carcinoma ontogeny and mechanisms of lethality. *Nature Reviews Nephrology*.

[2] Wettersten H. I., Aboud O. A., Lara P. N., Weiss R. H. (2017). Metabolic reprogramming in clear cell renal cell carcinoma. *Nature Reviews Nephrology*.

[3] Makhov P., Joshi S., Ghatalia P., Kutikov A., Uzzo R. G., Kolenko V. M. (2018). Resistance to systemic therapies in clear cell renal cell carcinoma: mechanisms and management strategies. *Molecular Cancer Therapeutics*.

[4] Ren X., Chen X., Ji Y. (2020). Upregulation of KIF20A promotes tumor proliferation and invasion in renal clear cell carcinoma and is associated with adverse clinical outcome. *Aging*.

[5] Eremina M., Rozhon W., Yang S., Poppenberger B. (2015). ENO2 activity is required for the development and reproductive success of plants, and is feedback-repressed by AtMBP-1. *The Plant journal : for cell and molecular biology*.

[6] Isgrò M. A., Bottoni P., Scatena R. (2015). Neuron-specific enolase as a biomarker: biochemical and clinical aspects. *Advances in Cancer Biomarkers*.

[7] Wang Q., Chen C., Ding Q. (2020). METTL3-mediated m6A modification of HDGF mRNA promotes gastric cancer progression and has prognostic significance. *Gut*.

[8] Liu C. C., Wang H., Wang W. D. (2018). ENO2 promotes cell proliferation, glycolysis, and glucocorticoid-resistance in acute lymphoblastic leukemia. *Cellular Physiology and Biochemistry: International Journal of Experimental Cellular Physiology, Biochemistry, and Pharmacology*.

[9] Zheng Y., Wu C., Yang J. (2020). Insulin-like growth factor 1-induced enolase 2 deacetylation by HDAC3 promotes metastasis of pancreatic cancer. *Signal Transduction and Targeted Therapy*.

[10] Tang C., Wang M., Dai Y., Wei X. (2021). Kruppel-like factor 12 suppresses bladder cancer growth through transcriptionally inhibition of enolase 2. *Gene*.

[11] Sun C., Liu M., Zhang W. (2021). Overexpression of enolase 2 is associated with worsened prognosis and increased glycikolysis in papillary renal cell carcinoma. *Journal of Cellular Physiology*.

[12] Wei Y., Chen X., Ren X. (2021). Identification of MX2 as a novel prognostic biomarker for sunitinib resistance in clear cell renal cell carcinoma. *Frontiers in Genetics*.

[13] Ganapathy-Kanniappan S., Geschwind J. F. (2013). Tumor glycolysis as a target for cancer therapy: progress and prospects. *Molecular Cancer*.

[14] Capitanio U., Bensalah K., Bex A. (2019). Epidemiology of Renal Cell Carcinoma. *European Urology*.

[15] Pastushenko I., Blanpain C. (2019). EMT transition states during tumor progression and metastasis. *Trends in Cell Biology*.

[16] Fang L., Zhang Y., Zang Y. (2019). HP-1 inhibits the progression of ccRCC and enhances sunitinib therapeutic effects by suppressing EMT. *Carbohydrate Polymers*.

[17] Gorka J., Marona P., Kwapisz O. (2021). MCPIP1 inhibits Wnt/*β*-catenin signaling pathway activity and modulates epithelial-mesenchymal transition during clear cell renal cell carcinoma progression by targeting miRNAs. *Oncogene*.

[18] Uprety D., Adjei A. A. (2020). KRAS: from undruggable to a druggable cancer target. *Cancer Treatment Reviews*.

[19] Waters A. M., Der C. J. (2018). KRAS: The critical driver and therapeutic target for pancreatic cancer. *Cold Spring Harbor Perspectives in Medicine*.

[20] Wang Y., Dong D., Jiang S. (2018). miR-216b post-transcriptionally downregulates oncogene KRAS and inhibits cell proliferation and invasion in clear cell renal cell carcinoma. *Cellular Physiology and Biochemistry: International Journal of Experimental Cellular Physiology, Biochemistry, and Pharmacology*.

[21] Viallard C., Larrivée B. (2017). Tumor angiogenesis and vascular normalization: alternative therapeutic targets. *Angiogenesis*.

[22] Cao J., Liu X., Yang Y. (2020). Decylubiquinone suppresses breast cancer growth and metastasis by inhibiting angiogenesis via the ROS/p53/ BAI1 signaling pathway. *Angiogenesis*.

[23] Wang G., Li H., Hou Y. (2022). LncRNA MAGI2-AS3 inhibits tumor progression and angiogenesis by regulating ACY1 via interacting with transcription factor HEY1 in clear cell renal cell carcinoma. *Cancer Gene Therapy*.

[24] Wang Q., Ding H., He Y. (2019). NLRC5 mediates cell proliferation, migration, and invasion by regulating the Wnt/*β*-catenin signalling pathway in clear cell renal cell carcinoma. *Cancer Letters*.

[25] Galluzzi L., Humeau J., Buqué A., Zitvogel L., Kroemer G. (2020). Immunostimulation with chemotherapy in the era of immune checkpoint inhibitors. *Nature Reviews Clinical Oncology*.

[26] Locati M., Curtale G., Mantovani A. (2020). Diversity, mechanisms, and significance of macrophage plasticity. *Annual Review of Pathology*.

[27] Xie Y., Chen Z., Zhong Q. (2021). M2 macrophages secrete CXCL13 to promote renal cell carcinoma migration, invasion, and EMT. *Cancer Cell International*.

[28] Martínez V. G., Rubio C., Martínez-Fernández M. (2017). BMP4 induces M2 macrophage polarization and favors tumor progression in bladder cancer. *Clinical Cancer Research*.

[29] Xu W., Liu W. R., Xu Y. (2021). Hexokinase 3 dysfunction promotes tumorigenesis and immune escape by upregulating monocyte/macrophage infiltration into the clear cell renal cell carcinoma microenvironment. *International Journal of Biological Sciences*.

[30] Fang Z., Sun Q., Yang H., Zheng J. (2021). SDHB suppresses the tumorigenesis and development of ccRCC by inhibiting glycolysis. *Frontiers in Oncology*.

[31] Chen Y., Yang L., Liu N. (2021). NONO-TFE3 fusion promotes aerobic glycolysis and angiogenesis by targeting HIF1A in NONO-TFE3 translocation renal cell carcinoma. *Current Cancer Drug Targets*.

