# Supplementary figures and images for: Integrated Analysis of the Role of Enolase 2 in Clear Cell Renal Cell Carcinoma
Source: Dis Markers. 2022 Nov 14;2022:6539203. doi: 10.1155/2022/6539203 (PMC9678487; doi:10.1155/2022/6539203)

Figure S1. Western blot was used to validate the knockdown efficiency of ENO2

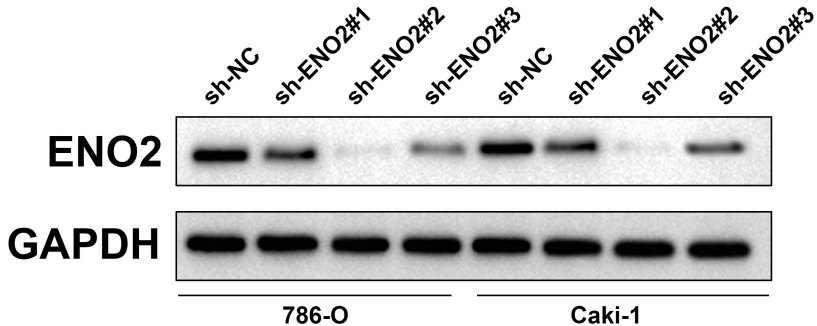

Supplement: Supplementary Materials — Figure S1. Western blot was used to validate the knockdown efficiency of ENO2. [file 6539203.f1.pdf]
